# Nesfatin-1 Regulates Feeding, Glucosensing and Lipid Metabolism in Rainbow Trout

**DOI:** 10.3389/fendo.2018.00484

**Published:** 2018-08-28

**Authors:** Ayelén M. Blanco, Cristina Velasco, Juan I. Bertucci, José L. Soengas, Suraj Unniappan

**Affiliations:** ^1^Laboratory of Integrative Neuroendocrinology, Department of Veterinary Biomedical Sciences, Western College of Veterinary Medicine, University of Saskatchewan, Saskatoon, SK, Canada; ^2^Laboratorio de Fisioloxía Animal, Departamento de Bioloxía Funcional e Ciencias da Saúde, Facultade de Bioloxía and Centro de Investigación Mariña, Universidade de Vigo, Vigo, Spain; ^3^Instituto de Investigaciones Biotecnológicas-Instituto Tecnológico Chascomús, Chascomús, Argentina

**Keywords:** NUCB2, nutrient sensing, hypothalamus, hindbrain, liver, fish

## Abstract

Nesfatin-1 is an 82 amino acid peptide that has been involved in a wide variety of physiological functions in both mammals and fish. This study aimed to elucidate the role of nesfatin-1 on rainbow trout food intake, and its putative effects on glucose and fatty acid sensing systems. Intracerebroventricular administration of 25 ng/g nesfatin-1 resulted in a significant inhibition of appetite, likely mediated by the activation of central POMC and CART. Nesfatin-1 stimulated the glucosensing machinery (changes in *sglt1, g6pase, gsase*, and *gnat3* mRNA expression) in the hindbrain and hypothalamus. Central fatty acid sensing mechanisms were unaltered by nesfatin-1, but this peptide altered the expression of mRNAs encoding factors regulating lipid metabolism (*fat/cd36, acly, mcd, fas, lpl, ppar*α, and *ppar*γ), suggesting that nesfatin-1 promotes lipid accumulation in neurons. In the liver, intracerebroventricular nesfatin-1 treatment resulted in decreased capacity for glucose use and lipogenesis, and increased the potential of fatty acid oxidation. Altogether, the present results demonstrate that nesfatin-1 is involved in the homeostatic regulation of food intake and metabolism in fish.

## Introduction

Food intake in vertebrates is subject to a complex regulation involving both peripheral components in charge of transmitting the metabolic status of the organism, and the central nervous system (CNS), responsible for receiving, processing and responding to this information (1). Specific hypothalamic nuclei in the CNS are responsive to changes in energy status, and respond with variations in the expression of key neuropeptides (particularly, agouti-related protein (AgRP)/neuropeptide Y (NPY), and pro-opio melanocortin (POMC)/cocaine and amphetamine-related transcript (CART)), ultimately leading to changes in food intake (2). These hypothalamic neurons, together with neurons from the hindbrain possess nutrient sensing mechanisms able to detect the levels of nutrients, particularly glucose, fatty acids and amino acids, as demonstrated in mammals (2) and in fish (3, 4).

Glucosensing in fish is mainly driven by the canonical mechanism based on glucokinase (GCK), glucose facilitative transporter 2 (GLUT2) and ATP-dependent inward rectified potassium channel (K_ATP_) (3–6), although GCK-independent glucosensing mechanisms based on the sodium-glucose linked transporter 1 (SGLT-1), liver X receptor (LXR) and sweet taste receptor have been also described in the rainbow trout brain (7, 8). Fatty acid sensing mechanisms described so far in the fish brain are based on carnitine palmitoyltransferase 1 (CPT-1), fatty acid translocase (FAT/CD36), mitochondrial production of reactive oxygen species (ROS), and lipoprotein lipase (LPL) (9–11).

The mechanisms linking the function of nutrient sensing systems with changes in the expression of hypothalamic appetite-regulating neuropeptides are scarcely known in both mammals and fish. Studies available in mammals point to the activation of mechanistic target of rapamycin (mTOR) and the inhibition of AMP-activated protein kinase (AMPK) as mediators between the activation of nutrient sensing systems and modulation of neuropeptides expression. This would occur through the modulation of the transcription factors forkhead box protein O1 (FOXO1), cAMP response element binding protein (CREB), and brain homeobox transcription factor (BSX) (12–14). In fish, available evidence suggests that comparable signaling pathways and transcription factors might be linking nutrient sensing and the expression of neuropeptides (15–17).

Central nutrient sensing mechanisms have been described to be subject of endocrine modulation in mammals (2, 18). In fish, it was reported that the glucosensor mechanism dependent on GCK-GLUT2-K_ATP_ is modulated at the hypothalamic level by insulin (19), leptin (20), ghrelin (21), cholecystokinin (22), and glucagon-like peptide 1 (23). Insulin (24) and ghrelin (25) have been also found to modulate fatty acid sensing mechanisms.

Nesfatin-1 is an 82 amino acid peptide encoded in the N-terminal region of nucleobindin-2 (NUCB2) gene (26). It is mainly expressed in the brain, particularly hypothalamus, although it is also present in a wide variety of peripheral organs (27–30). Specific binding sites for nesfatin-1 were detected both in the CNS and peripheral organs (31). Nesfatin-1 has been mainly studied for its inhibitory action on food intake in both mammals (32–34) and fish (35, 36), but it has also been implicated in the regulation of cardiac functions (37, 38), lipid metabolism (39, 40), glucose homeostasis (33, 41, 42), and reproductive functions (43–45), among others. Available studies in mammals have shown that a subpopulation of NUCB2/nesfatin-1-expressing neurons is activated in the rat brain in response to hypoglycemia (46), and that nesfatin-1 modulates the excitability of glucosensing neurons in the rat brain (34, 47, 48). Furthermore, nesfatin-1 has been reported to be modulated by nutrients in mammals (49) and fish (50, 51). However, as far as we are concerned, the role of nesfatin-1 as a modulator of nutrient sensing systems has not been studied to date in fish.

Therefore, this study first aimed to assess if nesfatin-1 intracerebroventricular (ICV) treatment is anorectic in rainbow trout in a way similar to other mammalian and fish species. Once demonstrated, we aimed to determine the putative effects of nesfatin-1 on the mechanisms involved in glucose and fatty acid sensing in brain regions implicated in the homeostatic regulation of food intake, i.e., hypothalamus and hindbrain (1, 4). For this, nesfatin-1 was ICV injected and the hypothalamic and hindbrain expression of mRNAs involved in (i) appetite-regulating neuropeptides (*npy, agrp, pomca1, cart*); (ii) glucosensing and glucose metabolism: *glut2, sglt1, gck, pyruvate-kinase* (*pk*), *glucose 6-phosphatase* (*g6pase*), *phosphoenolpyruvate carboxykinase* (*pepck*), *glycogen synthase* (*gsase*), *guanine nucleotide-binding protein G(t) subunit alpha transducing 3* (*gnat3*); (iii) fatty acid sensing and metabolism: *fat*/*cd36, ATP-citrate lyase* (*acly*), *malonyl-CoA decarboxylase* (*mcd*), *fatty acid synthase* (*fas*), *cpt-1a/b/c, citrate synthase* (*cs*), *lpl, peroxisome proliferator-activated receptor type alpha and gamma* (*ppar*α, *ppar*γ), *sterol regulatory element-binding protein type 1c* (*srebp1c*); and iv) mitochondrial activity: *uncoupling protein 2a* (*ucp2a)* and *inward-rectifier K channel pore type 6.x-like, kir6.x-like*), was quantified. We also evaluated the phosphorylation status of the proteins mTOR, AMPKα, CREB, and FOXO1. The impact of the ICV treatment with nesfatin-1 was also assessed in the liver since this is the main tissue involved in energy homeostasis. Additionally, hypothalamic sensing of nutrients is known to induce changes in liver metabolism through sympathetic outflow, as described in mammals (2) and fish (1).

## Materials and methods

### Animals

Female rainbow trout (*Oncorhynchus mykiss*), with a body weight (bw) of 30 ± 5 g, were obtained from a local commercial supplier. Fish were housed in 700 L aquaria with filtered fresh water at 13 ± 1°C and continuous aeration, and maintained under a 12 h light:12 h darkness (12L:12D) photoperiod (lights on at 07:00 h). Food from a commercial pellet diet specifically designed for salmonids (Ewos Pacific 3 mm, Fish Farm Supply Co, Surrey, Canada) was offered daily at 10:00 h until visual apparent satiety. All studies adhered to the Canadian Council of Animal Care guidelines, and were approved by the Animal Research Ethics Board of the University of Saskatchewan (Protocol Number 2012-0082).

### Experimental designs

#### Effect of nesfatin-1 on rainbow trout food intake

Following a 3-week acclimation period, fish were divided into two experimental groups, control and experimental (*n* = 5 fish/group). Food intake was registered during 1 week before treatment to evaluate basal levels of food intake. For this, a pre-weighed amount of food was offered to fish in each tank. After feeding, the uneaten food was collected, dried and weighed. The amount of food consumed by all fish in each tank was calculated as previously described as the difference from the feed offered (52, 53). On the day of experiment, fish were lightly anesthetized with tricaine methanesulfonate (TMS-222, Syndel Laboratories, Canada), weighed and ICV injected with either saline alone (control group) or containing 25 ng/g of goldfish nesfatin-1 (VPISIDKTKVKLPEETVKESPQNVDTGLHYDRYLREVIDFLEKDQHFREKLHNTDMEDIKQGKLAKELDFVSHHVRTK LDEL; GenScript, Piscataway, USA; experimental group). ICV injections were performed in the third ventricle as previously described (54). Dose of nesfatin-1 used here was chosen based on our previous experiments in goldfish (35, 36). After injections, fish were returned to their corresponding tanks and allowed to recover. Food was recovered at 2, 6, and 24 h post-injection, and food intake was quantified as described above. The experiment was repeated three times, and results shown correspond to the mean of the three experiments.

#### Effect of nesfatin-1 on glucose and fatty acid sensing systems in rainbow trout

Following a 3-week acclimation period, fish were divided into two experimental groups, control and experimental (*n* = 9 fish/group). On the day of experiment, fish were ICV injected with either saline alone (control group) or containing 25 ng/g nesfatin-1 (experimental group), as described in Section Effect of nesfatin-1 on rainbow trout food intake. After 2 h, fish were anesthetized again, sacrificed by decapitation, and samples of hypothalamus, hindbrain and liver were collected, frozen in liquid nitrogen and stored at −80°C until analysis.

### Quantification of mRNA abundance by real-time quantitative PCR (RT-qPCR)

Total RNA from hypothalamus, hindbrain and liver was isolated using Ribozol RNA Extraction Reagent (aMReSCO, Toronto, Canada) and treated with DNAse. RNA purity was validated by optical density (OD) absorption ratio (OD 260/280 nm) using a NanoDrop 2000c (Thermo, Vantaa, Finland). Then, an aliquot of 1 μg of total RNA was reverse transcribed into cDNA in a 20 μL reaction volume using iScript Reverse Transcription Supermix for RT-qPCR (Bio-Rad, Mississauga, Canada) according to the manufacturer's instructions. Real-time quantitative PCRs were performed using iQ SYBR Green Supermix (Bio-Rad). The specific primer sequences used are shown in Table 1 and were ordered from IDT (Toronto, Canada). Primers used in this study were previously validated in rainbow trout (8, 25). Genes were amplified in duplicated RT-qPCR runs using a 96-well plate loaded with 1 μL of cDNA and 5 10^−7^ M of each forward and reverse primer in a final volume of 10 μL. RT-qPCR cycling conditions consisted of an initial step of 95°C for 3 min, and 35 cycles of 95°C for 10 s and specific annealing and extension temperature (Table 1) for 30 s. A melting curve was systematically monitored (temperature gradient at 0.5°C/5 s from 65 to 95°C) at the end of each run to confirm specificity of the amplification reaction. Wells containing RNA samples and water instead of cDNA were run for each reaction as negative controls. Only efficiency values between 85 and 100% were accepted (*R*^2^ for all genes assessed were always higher than 0.95). All runs were performed using a CFX Connect Real-Time System (Bio-Rad). The 2-ΔΔCt method (55) was used to determine the relative mRNA expression in the different samples. Relative quantification of target gene transcripts was done using β-actin gene expression as reference, which was stably expressed in this experiment. The mRNA expression in the control was considered to be 1, and the expression in experimental fish was calculated as a fold of the expression level in controls.

**Table 1:** Primers used for quantifying gene expression by RT-qPCR in this study.

**Gene**	**Data base**	**Accession number**	**Forward primer (5′-3′)**	**Reverse primer (5′-3′)**	**Annealing temperature (°C)**
*β-actin*	GenBank	NM_ 001124235.1	GATGGGCCAGAAAGACAGCTA	TCGTCCCAGTTGGTGACGAT	59
*acly*	GenBank	CA349411.1	CTGAAGCCCAGACAAGGAAG	CAGATTGGAGGCCAAGATGT	60
*agrp*	GenBank	CR376289	ACCAGCAGTCCTGTCTGGGTAA	AGTAGCAGATGGAGCCGAACA	60
*cart*	GenBank	NM_001124627	ACCATGGAGAGCTCCAG	GCGCACTGCTCTCCAA	60
*cpt-1a*	GenBank	AF327058	TCGATTTTCAAGGGTCTTCG	CACAACGATCAGCAAACTGG	55
*cpt-1b*	GenBank	AF606076	CCCTAAGCAAAAAGGGTCTTCA	CATGATGTCACTCCCGACAG	55
*cpt-1c*	GenBank	AJ619768	CGCTTCAAGAATGGGGTGAT	CAACCACCTGCTGTTTCTCA	59
*cs*	Tigr	TC89195	GGCCAAGTACTGGGAGTTCA	CTCATGGTCACTGTGGATGG	55
*fas*	Sigenae	tcab0001c.e.065.1.s.om.8	GAGACCTAGTGGAGGCTGTC	TCTTGTTGATGGTGAGCTGT	59
*fat/cd36*	DFCI	AY606034.1	CAAGTCAGCGACAAACCAGA	ACTTCTGAGCCTCCACAGGA	62
*g6pase*	Sigenae	cay0019b.d.18_3.1.s.om.8.1-1693	CTCAGTGGCGACAGAAAGG	TACACAGCAGCATCCAGAGC	55
*gck*	GenBank	AF053331	GCACGGCTGAGATGCTCTTTG	GCCTTGAACCCTTTGGTCCAG	60
*glut2*	GenBank	AF321816	GTGGAGAAGGAGGCGCAAGT	GCCACCGACACCATGGTAAA	59
*gnat3*	Sigenae	CU073912	GCAAGACGTGCTGAGGACCA	ATGGCGGTGACTCCCTCAAA	60
*gsase*	GenBank	BT073381.1	CGTGGTGAGAGGAAGGAACTGAGC	CCGTTGAGACCGTGGAGACA	59
*kir6.x-like*	Sigenae	CA346261.1.s.om.8:1:773:1	TTGGCTCCTCTTCGCCATGT	AAAGCCGATGGTCACCTGGA	60
*leptin*	GenBank	AB354909.1	GCGGGAGCTTCACTCTCATT	GAGGTCTGCCCAGTCTAGGA	60
*lpl*	GenBank	AJ224693	TAATTGGCTGCAGAAAACAC	CGTCAGCAAACTCAAAGGT	59
*mcd*	Sigenae	BX869708.s.om.10	TCAGCCAGTACGAAGCTGTG	CTCACATCCTCCTCCGAGTC	60
*npy*	GenBank	NM_001124266	CTCGTCTGGACCTTTATATGC	GTTCATCATATCTGGACTGTG	58
*pepck*	GenBank	AF246149	GTTGGTGCTAAAGGGCACAC	CCCGTCTTCTGATAAGTCCAA	59
*pk*	GenBank	AF246146	CCATCGTCGCGGTAACAAGA	ACATAGGAAAGGCCAGGGGC	59
*pomca1*	Tigr	TC86162	CTCGCTGTCAAGACCTCAACTCT	GAGTTGGGTTGGAGATGGACCTC	60
*pparα*	GenBank	AY494835	CTGGAGCTGGATGACAGTGA	GGCAAGTTTTTGCAGCAGAT	55
*pparγ*	DFCI	CA345564	GACGGCGGGTCAGTACTTTA	ATGCTCTTGGCGAACTCTGT	60
*sglt1*	GenBank	AY210436	GGGCTGAACATCTACCTTGCT	CTCATAACCTCCCACCTCATTG	59
*srebp1c*	GenBank	CA048941.1	GACAAGGTGGTCCAGTTGCT	CACACGTTAGTCCGCATCAC	60
*ucp2a*	GenBank	DQ295324	TCCGGCTACAGATCCAGG	CTCTCCACAGACCACGCA	57

*ACLY, ATP-citrate lyase; AgRP, agouti-related peptide; CART, cocaine- and amphetamine-related transcript; CPT-1, carnitine palmitoyl transferase type 1; CS, citrate synthase; FAS, fatty acid synthetase; FAT/CD36, fatty acid translocase; G6Pase, glucose 6-phosphatase; GCK, glucokinase; GLUT2, glucose facilitative transporter 2; GNAT3, guanine nucleotide-binding protein G(t) subunit alpha transducing 3; GSase, glycogen synthase; Kir6.x-like, inward-rectifier K channel pore type 6.x-like; LPL, Lipoprotein lipase; MCD, malonyl-CoA decarboxylase; NPY, neuropeptide Y; PEPCK, phosphoenolpyruvate carboxykinase; PK, pyruvate-kinase; POMC-A1, pro-opiomelanocortin A1; PPARα, peroxisome proliferator activated receptor type a; PPARγ, peroxisome proliferator activated receptor type γ; SGLT1, sodium-glucose linked transporter 1; SREBP1c, sterol regulatory element-binding protein type 1c; UCP2a, mitochondrial uncoupling protein 2a*.

### Analysis of protein levels by western blot

Tissue samples (*n* = 3 fish) were homogenized in T-PER tissue protein extraction reagent (Thermo Fisher Scientific, Waltham, USA), and proteins were extracted according to the manufacturer's instructions and quantified by Bradford assay. Western blot protocol was performed as previously described (56). The samples (containing 20 μg protein) were prepared in 1x Laemmli buffer containing 0.2% 2-mercaptoethanol (Bio-Rad) and boiled at 95°C for 10 min. Then, the whole sample volume was electrophoresed in 8–16% Mini-PROTEAN® TGX™ precast protein gel (Bio-Rad). Precision plus protein™ Dual Color Standards (Bio-Rad) was used as molecular weight marker. Following electrophoresis, proteins were transferred to a 0.2 μm pore-size nitrocellulose membrane (Bio-Rad) using the Trans-Blot® Turbo™ transfer system (Bio-Rad), and membrane was blocked in 1x RapidBlock™ solution (aMReSCO). Then, membranes were incubated overnight with specific primary antibody, all obtained from Cell Signaling Technology (Danvers, USA) except otherwise specified: 1:500 anti-phospho AMPKα (Thr-172), 1:500 anti-AMPK, 1:500 anti-phospho CREB (Ser-133), 1:500 anti-CREB (48h2), 1:250 anti-phospho-FoxO1 (Thr-24), 1:250 anti-FoxO1 (L27), 1:500 anti-phospho-mTOR (Ser-2448), and 1:1000 anti-vinculin (Abcam, Toronto, ON, Canada). All these antibodies cross-react successfully with rainbow trout proteins of interest (57, 58). After washing, membranes were incubated with goat anti-rabbit IgG (H+L) HRP conjugate (Bio-Rad) diluted 1:2,000. For protein visualization the membrane was incubated in Clarity™ Western ECL substrate (Bio-Rad) and imaged using ChemiDoc™ MP imaging system (Bio-Rad) with chemiluminescence detection. Protein bands were quantified by densitometry using Image Lab software. Target protein content in each of the samples was calculated as the ratio between the phosphorylated and the total amount of protein, except for the case of mTOR for which phosphorylated levels were normalized to the vinculin content.

### Statistics

Statistical differences in mRNA and protein expression were assessed using *t*-test, after data were checked for normality and homogeneity of variance. Data that failed one of these requirements were log-transformed and re-checked. Significance was assigned when *p* < 0.05. All analyses were carried out using SigmaPlot version 12.0 (Systat Software Inc., San Jose, USA) statistics package.

## Results

### Nesfatin-1 decreases food intake in rainbow trout

**Figure 1:**
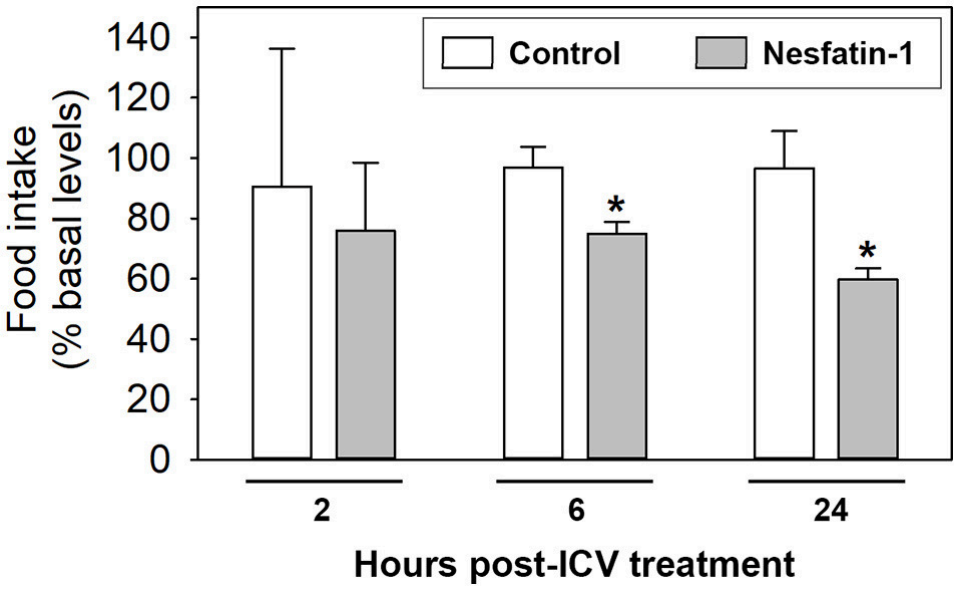
Food intake in rainbow trout 2, 6, and 24 h after intracerebroventricular administration of 1 ml/100 g body mass of saline alone (Control) or containing 25 ng/g of goldfish nesfatin-1 (Nesfatin-1). Levels of food intake are represented as mean + SEM of the percentage of food ingested with respect to baseline levels (calculated as the average of food intake the 3 days previous to experiment). Results correspond to the mean + SEM of the results obtained in three different experiments. Asterisks denote significant differences between control and treated groups assessed by *t*-test (^*^p < 0.05).

ICV treatment with nesfatin-1 resulted in a significant decrease in food intake at 6 and 24 h post-injection when compared to the control groups. Reduction of feeding was about 22 and 36%, respectively. No significant differences in food intake were observed between saline and nesfatin-1-injected fish at 2 h post-injection (Figure 1).

### Nesfatin-1 upregulates anorexigenic neuropeptides in the rainbow trout brain

**Figure 2:**
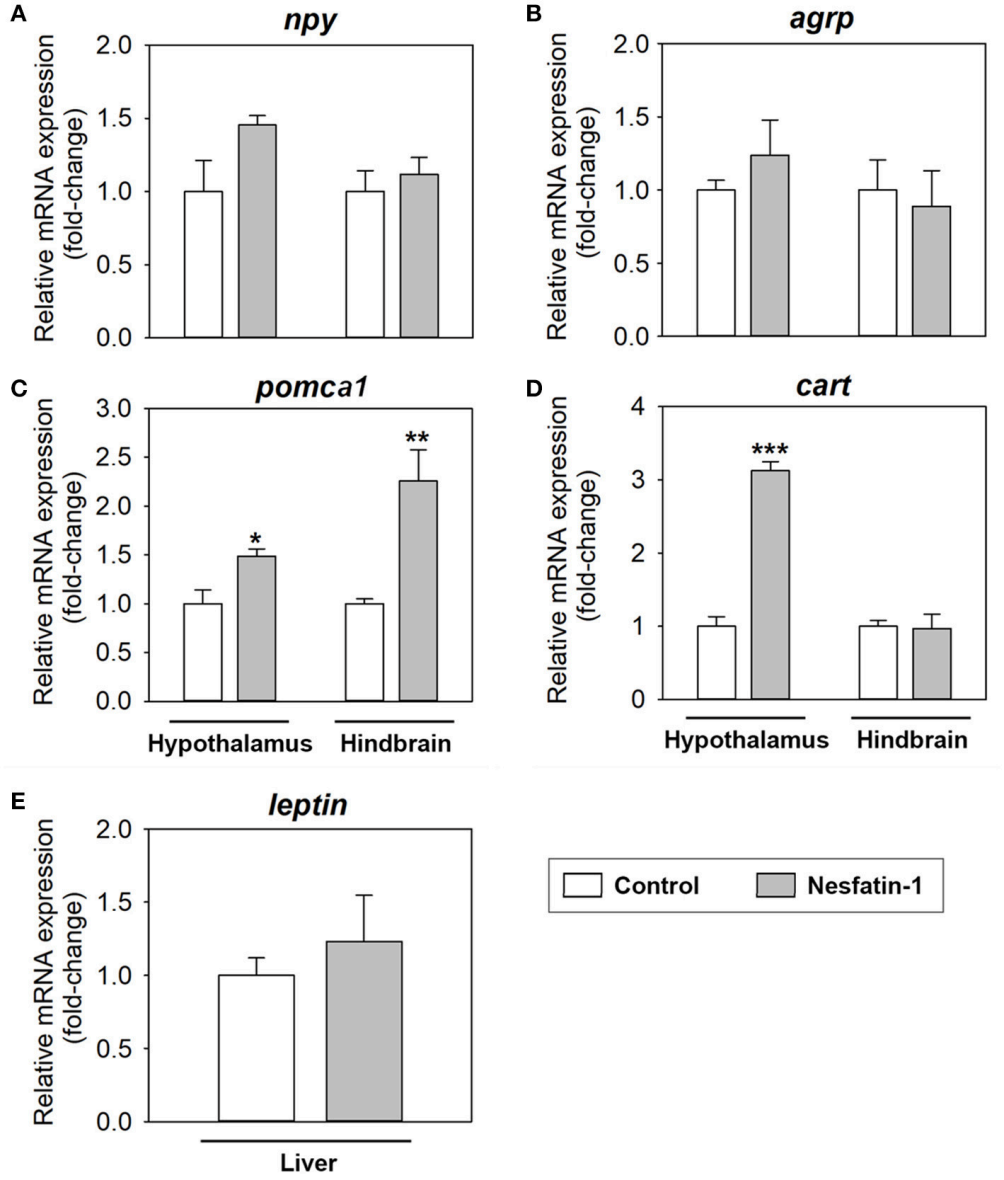
Effects of central administration of nesfatin-1 on the mRNA abundance of key appetite-regulating neuropeptides in the rainbow trout hypothalamus and hindbrain **(A–D)**, and of leptin in the rainbow trout liver **(E)**, at 2 h post-injection. Data obtained by RT-qPCR are shown as mean + SEM (*n* = 6 fish). Gene expression results are referred to control group and are normalized by β-actin expression. Asterisks denote significant differences between control and treated groups assessed by *t*-test (^*^p < 0.05, ^**^p < 0.01, ^***^p < 0.001).

ICV administration of nesfatin-1 resulted in a significant increase in the mRNA expression of *pomca1* in both the hypothalamus (1.5-fold) and hindbrain (2.3-fold), and of *cart* in the hypothalamus (3.3-fold), at 2 h post-injection (Figures 2C,D). Treatment with the hormone did not significantly alter the mRNA abundance of *npy* or *agrp* in neither the hypothalamus nor the hindbrain (Figures 2A,B), and of *leptin* in the liver (Figure 2E).

### Genes involved in glucosensing are affected by nesfatin-1 in rainbow trout

**Figure 3:**
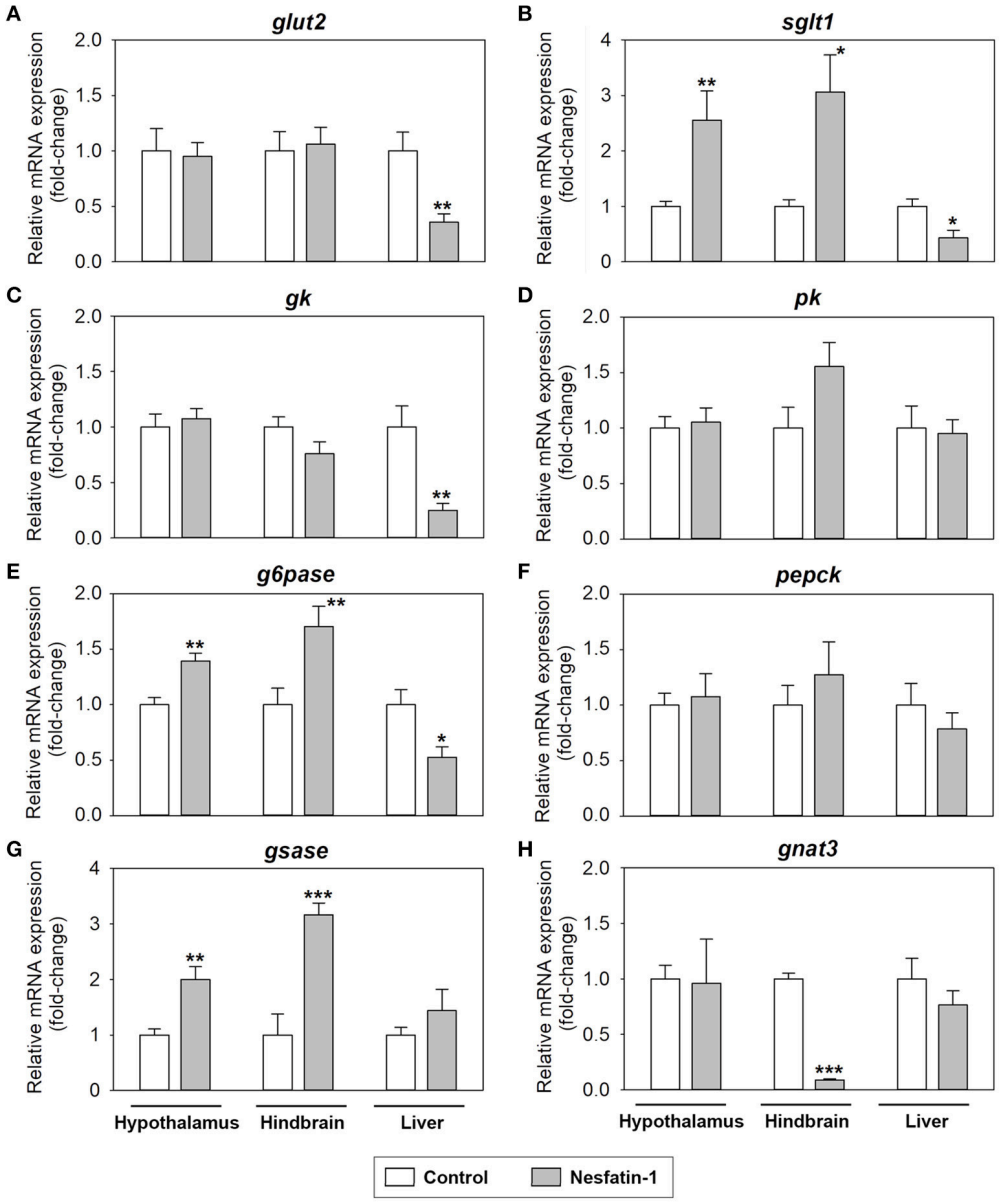
Effects of central administration of nesfatin-1 on the mRNA abundance of parameters related to glucosensing and glucose metabolism in the rainbow trout hypothalamus, hindbrain and liver at 2 h post-injection. **(A–H)** Data obtained by RT-qPCR are shown as mean + SEM (*n* = 6 fish). Gene expression results are referred to control group and are normalized by β-actin expression. Asterisks denote significant differences between control and treated groups assessed by *t*-test (^*^p < 0.05, ^**^p < 0.01, ^***^p < 0.001).

Changes in mRNA abundance of parameters related to glucosensing systems after nesfatin-1 treatment in the hypothalamus, hindbrain and liver of rainbow trout are shown in Figure 3. Nesfatin-1 was observed to produce a significant increase in the mRNA expression of *sglt1, g6pase*, and *gsase* in the hypothalamus and hindbrain at 2 h post-injection (Figures 3B,E,G). Additionally, gene expression of *gnat3* was downregulated in the hindbrain, but not in the hypothalamus, after ICV treatment with the hormone (Figure 3H). In the liver, nesfatin-1 was found to significantly decrease the mRNA levels of *glut2, sglt1, gck*, and *g6pase* (Figures 3A–C,E), with no changes in the remaining parameters analyzed.

### Nesfatin-1 modulates the expression of genes involved in lipid metabolism in rainbow trout

**Figure 4:**
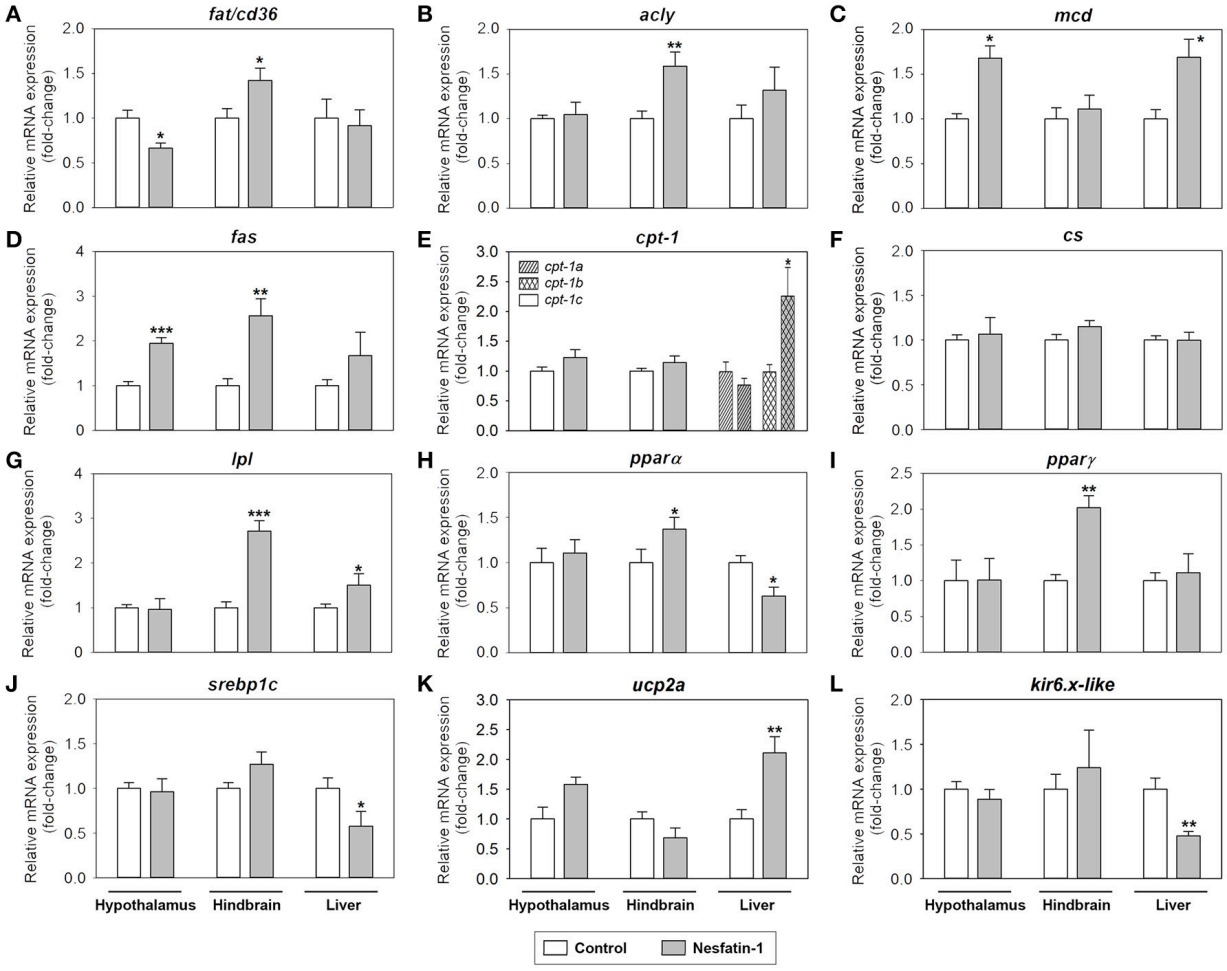
Effects of central administration of nesfatin-1 on the mRNA abundance of parameters related to fatty acid sensing and lipid metabolism **(A–J)** and mitochondrial activity **(K,L)** in the rainbow trout hypothalamus, hindbrain and liver at 2 h post-injection. Data obtained by RT-qPCR are shown as mean + SEM (*n* = 6 fish). Gene expression results are referred to control group and are normalized by β-actin expression. Asterisks denote significant differences between control and treated groups assessed by *t*-test (^*^p < 0.05, ^**^p < 0.01, ^***^p < 0.001).

Figure 4 shows the effects of nesfatin-1 on the mRNA expression of parameters related to fatty acid sensing, lipid metabolism and mitochondrial activity in the hypothalamus, hindbrain and liver of rainbow trout. Among the tissues studied, the most affected by ICV administration of nesfatin-1 was the hindbrain, where a significant increase in the gene expression of *fat/cd36, acly, fas, lpl, ppar*α, and *ppar*γ was detected at 2 h (Figures 4A,B,D,G–I). Nesfatin-1 treatment also led to a significant decrease in *fat/cd36* mRNAs and a significant increase in *mcd* and *fas* expression in the hypothalamus (Figures 4A,C). Finally, hepatic expression of *mcd, cpt-1b, lpl*, and *ucp2a* mRNAs were upregulated by nesfatin-1, while a significant downregulation of hepatic levels of *ppar*α, *srebp1c*, and *kir6.x-like* mRNAs was observed (Figures 4C,E,G,H,J–L). Expression of *cs* did not result altered after nesfatin-1 ICV treatment in any of the tissues studies (Figure 4F).

### Nesfatin-1 affects intracellular integrative pathways and transcription factors

**Figure 5:**
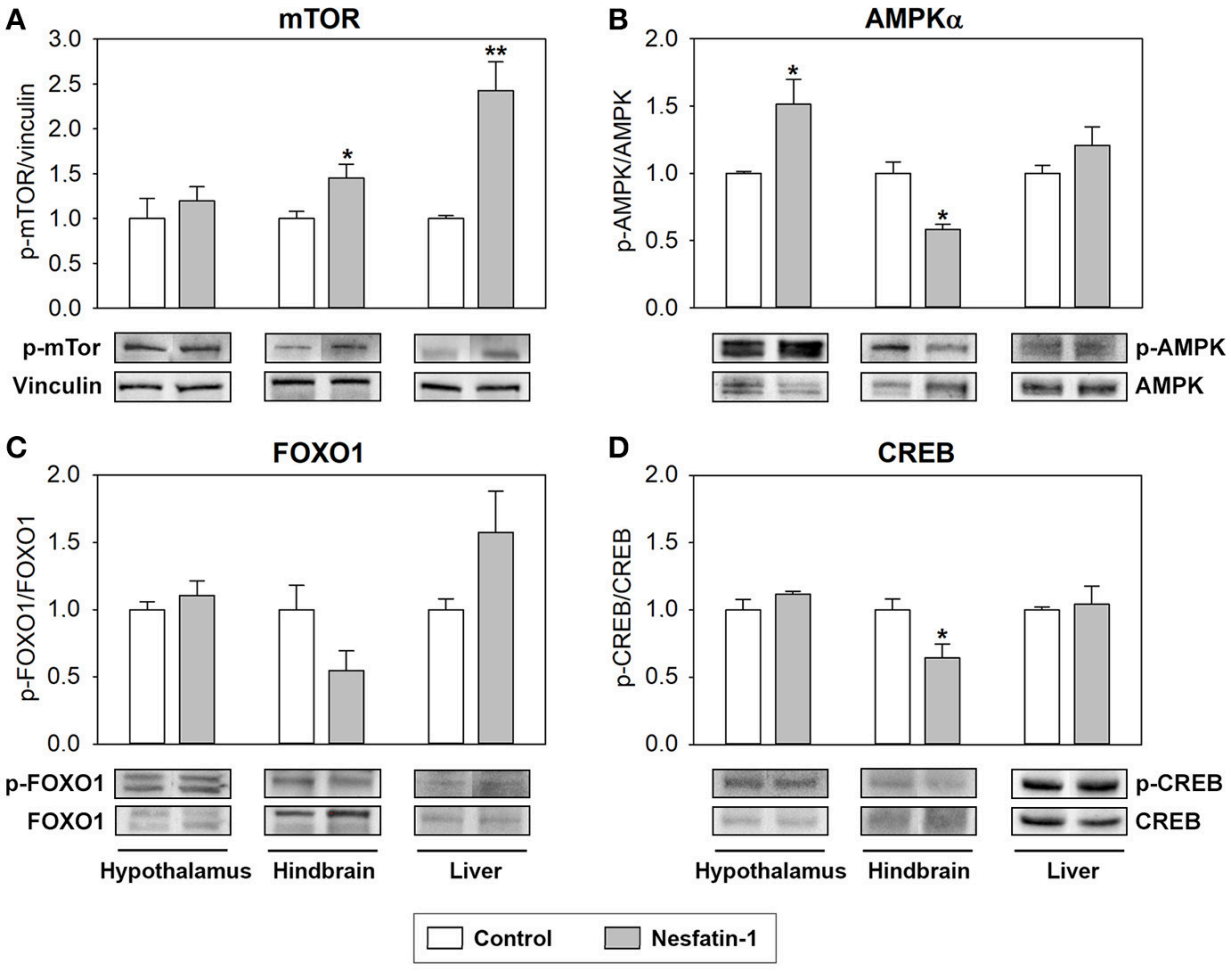
Effects of central administration of nesfatin-1 on the protein phosphorylation status of key intracellular integrative systems and transcription factors in the rainbow trout hypothalamus, hindbrain and liver at 2 h post-injection. **(A–D)** Western blots were performed on three individual samples per treatment, and a representative blot per treatment is shown here. Graphs represent the ratio between the phosphorylated protein and the total amount of the target protein, except for mTOR in which we used vinculin as reference. Asterisks denote significant differences between control and treated groups assessed by *t*-test (^*^p < 0.05, ^**^p < 0.01).

The effects of ICV administration of nesfatin-1 on the phosphorylation status of key intracellular integrative systems and transcription factors known to be involved in nutrient sensing mechanisms are shown in Figure 5. Nesfatin-1 was found to significantly increase the phosphorylation status of mTOR in the hypothalamus and hindbrain of rainbow trout (Figure 5A). The phosphorylation status of AMPKα and CREB was observed to be downregulated by nesfatin-1 in the hindbrain, while a significant upregulation of the phosphorylation status of AMPKα was detected in hypothalamus (Figures 5B,D). ICV administration of nesfatin-1 did not cause any significant change in the phosphorylation status of FOXO1 in any of the tissues studied (Figure 5C).

## Discussion

The present study evaluates the effects of ICV administered nesfatin-1 on food intake, and on the expression of key genes involved in nutrient sensing mechanisms in the brain and liver of rainbow trout.

### Nesfatin-1 is anorectic in rainbow trout

Our food intake experiment demonstrated that nesfatin-1 ICV treatment leads to a significant reduction in food intake levels in rainbow trout 6 and 24 h post-injection, in agreement with previous observations in other fish species [goldfish, (35, 36)], birds [chicks, (59)] and mammals [mice, (32, 60); rats, (26, 61)]. The upregulation of *pomca1* and *cart* mRNA expression in the trout brain, but the lack of effects on *npy* and *agrp* levels, suggests that the observed anorexigenic effect of nesfatin-1 in rainbow trout is mediated by the enhancement of potent brain appetite-inhibitors rather than by the suppression of central signals stimulating appetite. In mammals, nesfatin-1 inhibits NPY neurons causing their hyperpolarization (62), but has no effect on the mRNA expression of *Pomc* and *Cart* (32), in the hypothalamic arcuate nucleus. This might relate to the relative lower degree of co-localization between nesfatin-1 and NPY compared with that of nesfatin-1 and POMC (63, 64). On the other hand, a stimulatory effect of nesfatin-1 injection on the levels of mRNAs encoding *Pomc* and *Cart* has been observed in the mice hindbrain, specifically in the brainstem nucleus tractus solitarius (NTS) (32). Together, these observations suggest that the mechanisms underlying nesfatin-1 anorexigenic effects are similar in the hindbrain/NTS of fish and mammals, but not in the hypothalamus, where nesfatin-1 seems to modulate different neuronal populations, i.e., NPY neurons in mammals and POMC/CART neurons in fish. In favor of this hypothesis is the observed lack of changes in the brain mRNA expression of *kir6.x-like* in rainbow trout, considering that K_ATP_ channels are thought to mediate nesfatin-1-induced hyperpolarization of NPY neurons in the mammalian hypothalamus (62). Finally, we observed that nesfatin-1 anorexigenic action in rainbow trout seems to be independent of leptin signaling, since mRNA abundance of *leptin* in liver [the main site of synthesis in fish; (4)] did not change after nesfatin-1 treatment, in agreement with what is known in mammals (32, 65).

### Nesfatin-1 stimulates the glucosensing capacity in hindbrain and hypothalamus

Major findings of this study demonstrated that nesfain-1 modulates glucosensing in two brain regions (hypothalamus and hindbrain) known to be important in the regulation of glucose metabolism and homeostasis in fish (5, 66), and where nesfatin-1 binding sites have been previously described in mammals (31). Our results show that nesfatin-1 mainly activates glucosensing mechanisms in the rainbow trout brain, as demonstrated by the enhanced expression of *sglt1, g6pase*, and *gsase*, and the reduced expression of *gnat3* (only in hindbrain), results in general agreement with those observed in the same regions of rainbow trout subjected to glucose challenges (1, 3). This appears to be the first time in any vertebrates in which nesfatin-1 has been reported to modulate parameters involved in central glucosensing. In mammals, available studies addressed that nesfatin-1 treatment inhibits the firing rate of glucose-inhibited neurons and excites glucose-excitable neurons in the hindbrain (48) and hypothalamus (34, 47), both areas where the presence of nesfatin-1 binding sites have been characterized (67). Our results in rainbow trout demonstrated that the activation of the glucosensing capacity by nesfatin-1 was more marked in the hindbrain compared to the hypothalamus. Glucosensing capacity of the hindbrain is related to its indirect role in the hormonal regulation of food intake (via efferent pathways to the hypothalamus), and especially with its role in the homeostatic regulation of this process (68, 69). Thus, peripheral hormones responsive to food consumption and digestion modulate hunger and satiety mainly through hindbrain circuits (70), therefore supporting the here observed more remarkable response of the hindbrain glucosensing systems after the treatment with nesfatin-1. The less marked nesfatin-1-induced activation of the glucosensing capacity in the hypothalamus could relate to the reduced number of nesfatin-1 binding sites present in that area (47). Additionally, nesfatin-1 has been reported to increase insulin sensitivity in the rat brain through an increase in insulin receptor/insulin receptor substrate-1/AMPK/Akt/target of rapamycin complex (TORC) 2 phosphorylation (71). Results obtained here are in agreement with this action of nesfatin-1, which ultimately leads to an increase in glucose uptake and metabolism, and therefore a decrease in glucose circulating levels.

### Nesfatin-1 is not a modulator of fatty acid sensing

Present results indicate that nesfatin-1 is not a modulator of fatty acid sensing in the brain of the rainbow trout, as the mRNA expression of the parameters involved in fatty acid sensing analyzed did not respond to nesfatin-1 ICV treatment according to the anorexigenic nature of the peptide (1, 3). Thus, given that nesfatin-1 would be physiologically released when enough supply of lipids (and other energy sources) is in the body (67), it would make sense that central fatty acid sensing systems respond with an increase in the rate of entry of fatty acids into the cells and a decrease in the fatty acid synthesis pathways (1, 3). This hypothesis does not go hand in hand with the reduced expression of the fatty acid translocase *fat/cd36* in the hypothalamus and the enhanced expression of the lipogenic enzymes *fas* and *acly*, the enzyme involved in lipid release *lpl* and the lipogenesis-related transcription factor *ppar*α in the hypothalamus and/or the hindbrain after nesfatin-1 treatment. Instead, these results suggest that nesfatin-1 treatment produces an increase in the amount of fatty acids in the brain cells (as suggested by the increased expression of *fat/cd36* and *lpl*) and in the lipogenic activity (increased expression of *fas, acly*, and *ppar*α), particularly in the hindbrain, which as indicated above is in agreement with the role of this central area in the homeostatic control of food intake and energy balance. This nesfatin-1-induced promotion of lipid anabolism rather than lipid catabolism might relate to an increase in the storage of lipids, a more important energy source for the fish brain compared to glucose (72). However, while a higher amount of fatty acids appears to be in the brain cells in response to nesfatin-1, the lack of significant changes in the mRNA expression of *cpt-1c* (in charge of transporting fatty acids into the mitochondria) and *ucp2a* (related to mitochondrial activity) points to the assumption that such an increase in the levels of fatty acids is not related to their β-oxidation in order to produce energy. This hypothesis seems to be of higher importance in the hindbrain, as it is also supported by the lack of changes in the mRNA levels of *mcd* (promotes the entrance of fatty acids into the mitochondria). This enzyme is however upregulated by nesfatin-1 in the hypothalamus, apparently supporting β-oxidation. It must be taken into consideration that only mRNA abundance was analyzed in the present study, and thus it cannot be discarded that nesfatin-1 is indeed promoting β-oxidation by modulating protein levels and/or enzymatic activity. It can be also hypothesized that nesfatin-1 is instead promoting the accumulation of fatty acids in the neurons to promote neuronal growth and/or to maintain cell membranes. This hypothesis is supported by the observed increase in the mRNA levels of *ppar*γ, reported to be involved in neuronal development and improvement of neuronal health (73). The present results provide clear evidence to implicate nesfatin-1 in lipid metabolism in the brain, and future studies are needed to further investigate this proposed role.

### Administration of nesfatin-1 into the brain elicits changes in liver energy metabolism

This study demonstrated that nesfatin-1 ICV treatment resulted in a decrease in the capacity of liver to use glucose, as revealed by the reduced expression of *glut2, sglt1, gck*, and *g6pase* compared to the control group. These results suggest that nesfatin-1 may act at central level to elicit changes in the hepatic metabolism (via the autonomic nervous system). In other terms, nesfatin-1 may regulate the function of peripheral organs through the modulation of neural activity in order to maintain homeostasis. In the context of this hypothesis, the signal that the liver might be receiving from the central circuitries after nesfatin-1 administration is that the levels of food intake are reduced and therefore it is not necessary to produce and/or metabolize glucose. Previous studies in mammals have shown that ICV administration of nesfatin-1 leads to a decrease in the hepatic mRNA and protein expression and enzymatic activity of PEPCK (gluconeogenic enzyme), while does not modify the expression or activity of GCK and G6Pase (71). Similarly, hypothalamic nesfatin-1/NUCB2 knockdown rats have been shown to have increased hepatic levels of G6Pase and PEPCK (74). These observations demonstrate that central action of nesfatin-1 results in the inhibition of hepatic gluconeogenesis in mammals. Results obtained in the present study suggest that the response model seems to be slightly different in the rainbow trout, where the gluconeogenic potential seems not to be affected by ICV treatment with nesfatin-1 (no changes in *pepck* mRNAs) but instead it appears to be an inhibition in the hepatic use of glucose (decreased expression of *glut2, sglt1, gck*, and *g6pase*). These differences could relate to the differences between the mammalian and fish systems in terms of glucose metabolism, and the relative intolerance to glucose in carnivorous fish such as the rainbow trout (5, 75).

Regarding fatty acids, the presence of nesfatin-1 in the brain appears to attenuate the lipogenic activity and to stimulate fatty acid oxidation in the rainbow trout liver, as suggested by increased mRNA levels of *cpt-1b, lpl, mcd* and the mitochondrial activity-related gene *ucp2a*, and a decrease in the expression of the lipogenesis-related transcriptional factors *ppar*α and *srebp1c*, after nesfatin-1 treatment. This is comparable to the direct effects of nesfatin-1 treatment in the mammalian liver (40), but, more interestingly, is again suggesting that afferent signals coming from the brain are informing the liver of the anorectic signal detected, which is translated into an inhibition of fuel production, in this case lipids, for use in peripheral tissues.

### Nesfatin-1 in the brain elicits changes in the phosphorylation status of proteins involved in intracellular signaling and transcription factors

Changes in these parameters were most abundant in the hindbrain, where glucosensing systems and parameters involved in lipid metabolism were most affected by nesfatin-1. In this brain area, nesfatin-1 treatment resulted in an increase in the phosphorylation ratio of mTOR, while a decrease occurred in the phosphorylation ratio of AMPKα and CREB. These changes are not consistent with the few studies available in mammals showing that nesfatin-1 treatment upregulates CREB in a neural cell line (76) and reduces the phosphorylation of mTOR in the dorsal motor nucleus of the vagus (77). However, they are similar to those observed when levels of glucose and/or fatty acids increase in fish (16, 78), a scenario that would match the expected effects for an anorectic signal such as nesfatin-1. In the hypothalamus, only phosphorylation status of AMPKα was affected by nesfatin-1 and in the opposite direction than in the hindbrain. It seems that the more attenuated response of the glucosensing system to nesfatin-1 in the hypothalamus compared to the hindbrain is not enough to trigger significant changes in intracellular signaling pathways and transcription factors in the hypothalamus. Finally, in the liver, ICV treatment with nesfatin-1 resulted in a significant increase in the phosphorylation status of mTOR, in accordance with previous observation in mammals also obtained after ICV treatment with nesfatin-1 (71). This activation of hepatic mTOR after nesfatin-1 ICV treatment can be related with triggering the less use of fuels in this tissue, as discussed above.

In summary, this study demonstrates an anorexigenic action for nesfatin-1 in rainbow trout, likely mediated by the activation of central anorexigens (i.e., POMC and CART), and shows for the first time a role for this peptide in the modulation of glucosensing mechanisms and lipid metabolism in central locations of this fish species. Overall, results obtained in the brain (hypothalamus and hindbrain) point out that nesfatin-1 might stimulate glucosensing systems. Nesfatin-1 also appears to increase the levels of fatty acids in the brain cells, and we hypothesized that this could relate to promoting neural development and/or helping in maintaining cell membranes. The more marked effects of nesfatin-1 in the hindbrain might relate to a higher presence of nesfatin-1-expressing neurons and nesfatin-1 binding sites in this area in mammals (48). The presence of nesfatin-1 in the brain also seems to produce a decrease in the use of glucose, and to attenuate the lipogenic activity and stimulate fatty acid oxidation in the liver, thus apparently modifying energy expenditure in agreement with the anorectic nature of nesfatin-1. Together, this study shows for the first time that nesfatin-1 has an important role in the homeostatic regulation of food intake and is likely involved in energy expenditure in fish. Further studies are required to delve into the knowledge of the proposed novel role for nesfatin-1 in lipid metabolism in the fish brain.

## Author contributions

AB conducted experiments, analyzed data, prepared manuscript. CV helped in conducting experiments. JB helped in conducting experiments. JS contributed to experimental design, assisted with data analysis and edited manuscript for submission. SU contributed to experimental design, provided funding, assisted with data analysis and edited manuscript for submission.

### Conflict of interest statement

The authors declare that the research was conducted in the absence of any commercial or financial relationships that could be construed as a potential conflict of interest.
